# Supplementary material for: Electrochemical Characterization of Mancozeb Degradation for Wastewater Treatment Using a Sensor Based on Poly (3,4-ethylenedioxythiophene) (PEDOT) Modified with Carbon Nanotubes and Gold Nanoparticles
Source: Polymers (Basel). 2019 Sep 4;11(9):1449. doi: 10.3390/polym11091449 (PMC6780876; doi:10.3390/polym11091449)
Supplement: Supplementary file 1 [file polymers-11-01449-s001.pdf]

polymers

MDPI

Supplementary Materials: Electrochemical Characterization of Mancozeb Degradation for Wastewater Treatment Using a Sensor Based on Poly (3,4-ethylenedioxythiophene) (PEDOT) Modified with Carbon Nanotubes and Gold Nanoparticles

Roy Zamora-Sequeira [1,*], Fernando Alvarado-Hidalgo [2], Diana Robles-Chaves [3], Giovanni Sáenz-Arce [4], Esteban D. Avendano-Soto [5], Andrés Sánchez-Kopper [6] and Ricardo Starbird-Perez [6]

Received: 11 July 2019; Accepted: 30 August 2019; Published: 4 September 2019

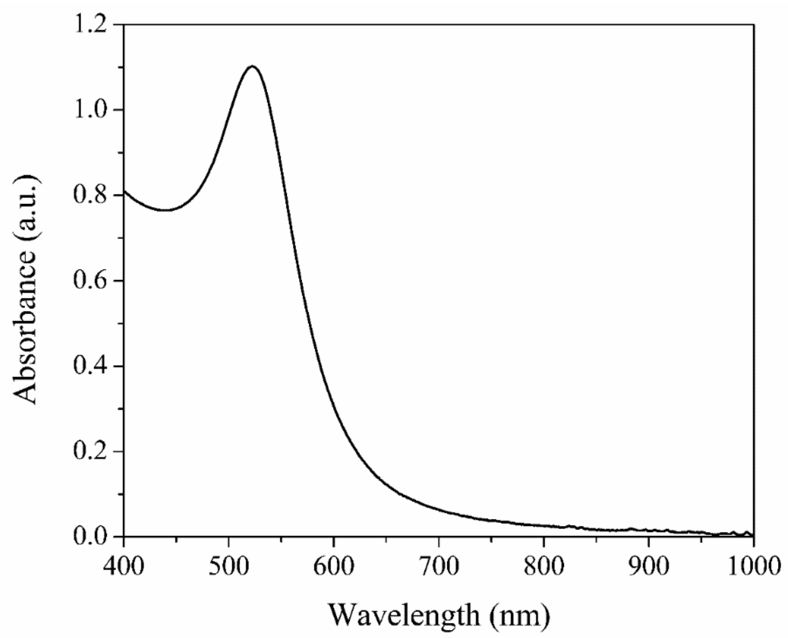

Figure 1. UV–vis absorption spectrum of gold nanoparticles.

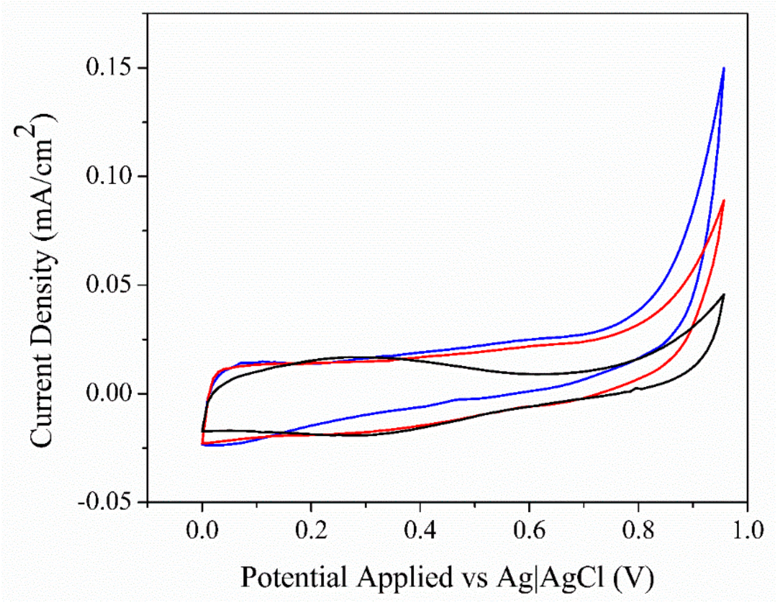

Figure 2. Cyclic voltammetry curves of aqueous solutions of MCZ. Blank (Black), 100 μM aqueous MCZ sample (Blue), and aqueous sample of 100 μM MCZ after treatment with UV light (Red).

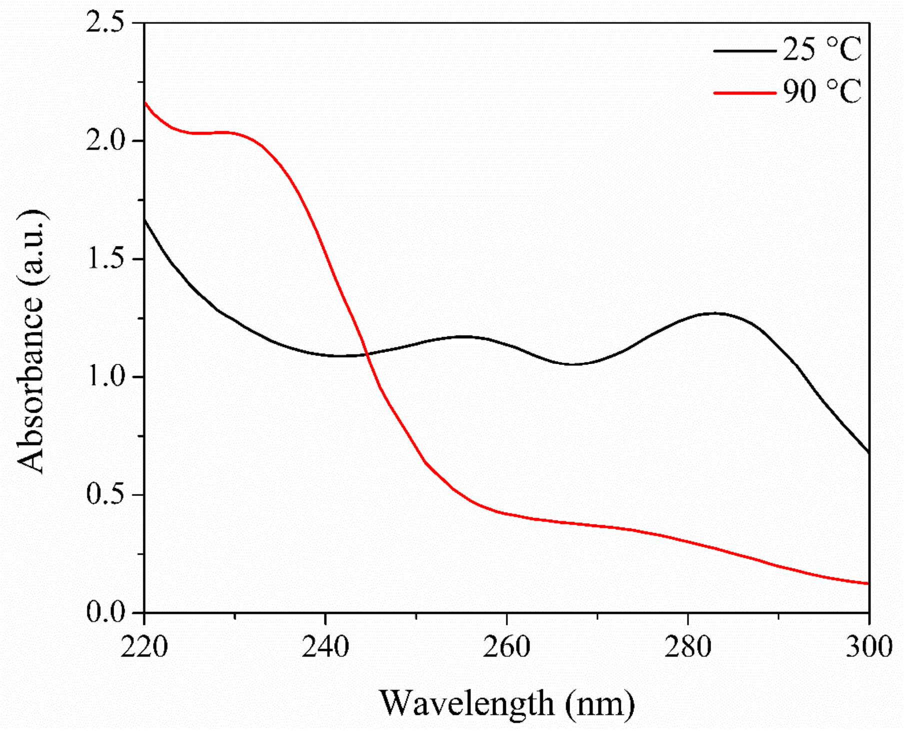

Figure 3. UV–vis spectra of mancozeb at 25 °C (Black) and 90 °C (Green).

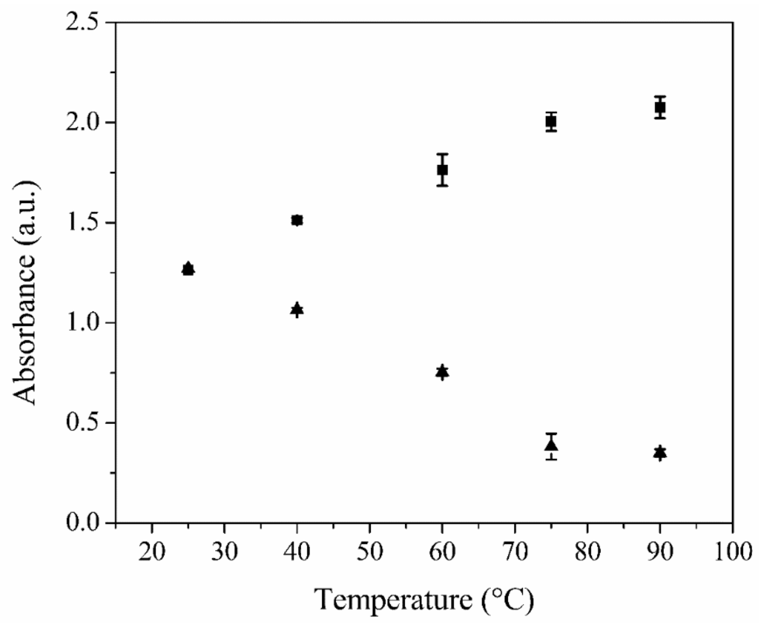

Figure 4. Increment and linear decrease absorbance vs temperature.

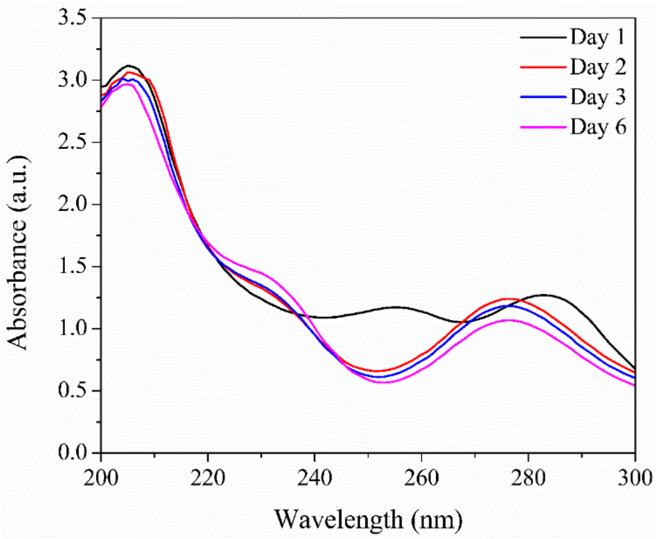

Figure 5. Plots of absorbance spectrums vs days at ambient temperature.

© 2019 by the authors. Submitted for possible open access publication under the terms and conditions of the Creative Commons Attribution (CC BY) license (http://creativecommons.org/licenses/by/4.0/).
